# Incidence and risk factors of chronic pain following hysterectomy among Southern Jiangsu Chinese Women

**DOI:** 10.1186/s12871-017-0394-3

**Published:** 2017-08-11

**Authors:** Chao Han, Zhijun Ge, Wenjie Jiang, Hailong Zhao, Tieliang Ma

**Affiliations:** The Affiliated Yixing Hospital of Jiangsu University, 75 Tongzhenguan Road, Yixing, Jiangsu 214200 People’s Republic of China

**Keywords:** Chronic post-surgical pain, Hysterectomy, Risk factors

## Abstract

**Background:**

Chronic post-surgical pain (CPSP) after hysterectomy has been recognized as a major clinical problem in the Western World. Reports on post-hysterectomy pain are relatively scarce in China. The aim of the current study was to prospectively investigate the incidence and the potential risk factors of CPSP at 3 months following hysterectomy in Chinese population.

**Methods:**

We assessed and collected data on preoperative socio-demographic characteristics, preexisting pain, anxiety and depression, sexual satisfaction, intra-operative variables, and acute postoperative pain intensity in a cohort of 870 women undergoing hysterectomy. The participants were interviewed to determine their suitability to diagnostic criteria of CPSP 3 months later. Logistic regression analyses were subsequently performed to identify predictors for CPSP.

**Results:**

The incidence of CPSP at 3 months after hysterectomy was 27.7%. Most of the women with CPSP suffered from mild pain and had a slight impact on daily life with sleep and emotion functional limitation. Risk factors for CPSP after hysterectomy were preoperative anxiety, depression, pelvic pain, preexisting pain, very-moderate sexual dissatisfaction, and acute postoperative pain at movement. Intra-operative dexmedetomidine infusion with 0.5 μg/kg/h was associated with a decreased incidence rate of chronic post-hysterectomy pain.

**Conclusion:**

Twenty-eight percent of patients after hysterectomy in southern Jiangsu china had CPSP with 92% of those women describing it as mild with sleep and emotion functional limitation. Patients with preoperative anxiety and depression, poor sexual satisfaction, preexisting pain, and acute postoperative pain on movement have been identified to be at risk to develop CPSP.

## Significance

Preoperative anxiety and depression, poor sex satisfaction, presence of pre-surgical pain elsewhere, and acute postoperative pain on movement are risk factors for CPSP in a Chinese population.

## Background

Chronic post-surgical pain (CPSP) is a major clinical problem which could lead to impaired physical function and reduced quality of life. Several studies have been published on development of CPSP following hysterectomy in western population, with the incidence being to be between 5 and 50% [1, 2]. The variability in rate of incidence might be due to different study designs and methodologies, selected samples, and CPSP definitions used in individual studies. In China, the annual incidence rate of hysterectomy has been reported to be as high as 80 per 100,000 women, with 250,000 procedures performed each year. This means a high prevalence of chronic post-hysterectomy pain in China. However, to date, there have been no reports of prevalence of CPSP following hysterectomy in Chinese population. Moreover, previous studies have demonstrated that differences in the genotype of individuals could influence the development of CPSP [3], indicating that ethnic difference might play an important role in occurrence of CPSP. The published incidence of CPSP in western women may therefore not apply to Chinese population. Above all, pertinent knowledge on incidence and risk factors that lead to development of CPSP after hysterectomy among Chinese women is essential for prevention and treatment of chronic post-hysterectomy pain in China. The primary aim of this study was to assess chronic pain 3 months after hysterectomy in a cohort of women in China. We also aim to elucidate the relative contribution of clinical and psychological risk factors for the development of CPSP following hysterectomy.

## Methods

### Design and study population

This prospective observational cohort study was approved by the ethics committee of the affiliated Yixing Hospital of Jiangsu University, China. Patients admitted for hysterectomy for benign indications from March 2014 to March 2016 in the affiliated Yixing Hospital of Jiangsu University were invited to participate in this study. Inclusion criteria: (1) age between 18 and 75 years, (2) able to understand consent procedures and questionnaire materials, (3) total or subtotal hysterectomy with or without oophorectomy. Patients who agreed to participate were explained about the procedures, and they signed the consent form. All participants were interviewed in person both preoperatively and 48 h postoperatively by a trained anesthetist. The follow-up interviews were completed 3 months after surgery. Exclusion criteria were cognitive impairment, a history of cancer, malignant uterine tumors, surgery-related infection.

### Preoperative questionnaire

Study-specific questionnaires were given to the patients for self-administration during the preoperative screening visit before surgery. The questionnaire consisted of questions on patients’ age, education, employment, body mass index (BMI), smoking, alcohol abuse, indications for hysterectomy, co-morbidities, and a history of caesarean section, laparotomy or laparoscopy.

Hospital Anxiety and Depression Scale (HADS) in a Chinese version was used for screening anxiety disorders and depression, considering the influence of psychological factors on chronic pain. HADS performed well in assessing symptom severity and in diagnosing anxiety disorders and depression in various populations, with significant internal consistency and concurrent validity [4]. A systematic review and meta-analysis of the association with preoperative anxiety and catastrophizing and Chronic Postsurgical Pain showed that there were no significant difference between HADS and other instruments such as STAI, ICD-9, MSPQ, MMPI [5]. HADS consists of an anxiety subscale (HAD-A) and a depression subscale (HAD-D), each of which contains 7 intermingled items, providing four answer options for each item ranging from 0 to 3. A cutoff threshold as 8 is identified of possibility of anxiety and depression disorders [6].

The Female Sexual Function Index (FSFI) is the most widely used instrument for female sexual health, comprising six domains: desire, arousal, lubrication, orgasm, satisfaction, and pain [7]. We assessed the degree of sexual satisfaction by means of the 14–16 items of FSFI in a Chinese version [8]. Three questions include how satisfied: (1) with amount of closeness with partner, (2) with sexual relationship, (3) with overall sex life. The score for each item ranges from 1 to 5. We identified a total score more than 12 as very-moderate satisfied, 8–12 as about equally satisfied and dissatisfied, and lower than 8 as very-moderate dissatisfied.

### Pain questionnaire

Assessment of pain and its consequences were performed by a trained anesthetist, in face-to-face interviews, before surgery, 48 h after surgery, and at 3 months after surgery. The Chinese version of brief pain inventory-short form (BPI-SF) was used to estimate the pain severity and the impact on daily life at every interview. The BPI-SF contained 11 items which references pain existed during the past 24 h. It was consisted of 2 subscales: pain severity (NRS: 4 items) and pain interference (7 items). Scores of NRS ranged from 0 to 10 (0 means no pain; 10 means worst pain imaginable).

### Surgical variables

A variety of methods including median lower abdominal approach, Pfannenstiel, vaginal, laparoscopic hysterectomy (LH), or laparoscopic assisted vaginal hysterectomy (LAVH), total or subtotal hysterectomy with or without oophorectomy have been used for removing the uterus and/or ovaries. Anesthetists determined the anesthesia protocol according to the surgery and custom, involving total intravenous anesthesia (TIVA), inhalation anesthesia (IA), epidural anesthesia. Intra-operative sedative and analgesic drugs, duration of operation, blood loss were collected. Postoperative analgesia and complications were also recorded from the hospital database.

### Follow-up during 48 h after surgery

An experienced anesthetist, who was in charge of pain questionnaire, visited patients within 48 h after hysterectomy. Pain related data were collected using BPI-SF, including the NRS value at rest and at movement.

### Follow-up at 3 months after surgery

BPI-SF was completed, when patients return to hospital at 3 months after hysterectomy. If diagnosed of CPSP, a douleur neuropathic 4-questionnaire (DN-4) was followed to ascertain whether the pain was a neuropathic Pain (NP). DN-4 is an instrument evaluating pain characteristics through 10 items, total score being 10 [9, 10]. If the patient score is ≥ 4, neuropathic pain is diagnosed.

### Definition of CPSP

We defined CPSP following hysterectomy based on the diagnosis criteria from the International Association for the Study of Pain (IASP) [11] and proposed by Macrae [12]: (1) pain development after a surgical procedure, (2) pain persisting for more than 3 months, (3) other causes for the pain should be excluded, and d) pain from a pre-existing problem should be explored and exclusion attempted.

### Statistical analyses

SPSS (version 18; Chicago, IL) was used to analyse all data. Categorical data were presented as numbers and percentages. Chi-square tests (χ^2^) were used to analyse socio-demographic, clinical, and psychological measures. Univariate logistic regression analysis was performed to test the influence of possible risk factors on CPSP at 3 months after surgery, and candidate covariates were chosen based on statistical significance or possible clinical importance. Only covariates with *P*-values less than 0.25 in the univariate analysis were entered in the multivariate model. Then, multiple logistic regression analyses were used to determine risk factors for CPSP. Two-sided *P*-values of 0.05 were considered statistically significant.

## Results

**Fig. 1:**
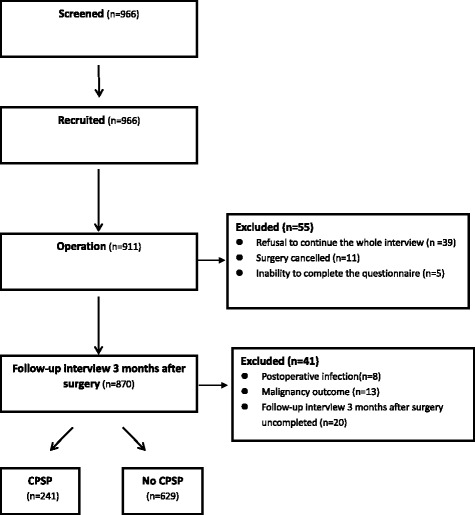
Selection of patients taken part in this study

From March 2014 to March 2016, 966 patients were recruited in the study. Of the 966 patients, 55(11 + 5 + 39) were excluded after surgery for the following reasons: (1) surgery cancelled, (2) inability to complete the questionnaire because of postoperative complications or intensive care unit admission, and (3) refusal to continue the whole interview; and another 41(13 + 8 + 20) were deleted because of: (1) Malignancy outcome, (2) postoperative infection, and (3) follow-up interview 3 months after surgery uncompleted. A total of 870 patients were finally enrolled in the analysis. According to the study definition, of 870 patients, 241 (27.7%) have been found to have CPSP and the remaining patients (629/870 -72.3%) were free of CPSP 3 months after hysterectomy. Patient inclusion is illustrated in the flow chart in Fig. 1.

Table 1 shows the Characteristics of pain and effect on daily life at 3 months after hysterectomy. 92.1% (222 of 241) women with CPSP reported mild pain and one tenth of the CPSP patients had a negative impact on daily life, mainly on mood and sleep. The most common pain located in pelvic region (46.9%, 113 of 241) and abdominal scars (35.7%, 86 of 241). Of 241 patients, 19 (7.9%, NRS ≥ 4) were reported moderate to severe pain, 85.5% (206/241) of the patients (206/241) reported to be experiencing pain less than once a week. Only 22 of 241 (9.1%) patients reported analgesic intake. Of 241 patients, 114 (47.3%) presented the characteristics of neuropathic pain with DN-4 score ≥ 4.

**Table 1:** Characteristics of pain and impact on daily life at 3 months following hysterectomy

	*N = 241*
Location	
Pelvic region	113 (46.9%)
Area of incision	86 (35.7%)
Vagina	19 (7.9%)
Lower back	14 (5.8%)
elsewhere	9 (3.7%)
Frequency	
Constantly	2 (0.8%)
Daily	2 (0.8%)
Several times a week	9 (3.7%)
once a week	22 (9.1%)
Less than once a week	206 (85.5%)
Pain severity (NRS 0–10)	
Mild pain (NRS 0–3)	222(92.1%)
Moderate pain (NRS 4–7)	17(7.1%)
Severe pain (NRS 7–10)	2(0.8%)
DN-4 total score	
< 4	127 (52.7%)
≥ 4	114 (47.3%)
Analgesic required	37 (15.4%)
Paracetamol	28 (11.6%)
NSAIDs	9 (3.7%)
Negative impact on daily life	
General activity	16 (6.6%)
Mood	38 (15.8%
Walking ability	15 (6.2)
Normal work	8 (3.3%)
Relations with other people	22 (9.1%)
Sleep	31 (12.9%)
Enjoyment of life	27 (11.2%)

Table 2 presents socio-demographic, clinical, preoperative psychological state, and sexual satisfaction variables for women with and without chronic pain at 3 months after hysterectomy. Factor with a statistically significant association with CPSP included preexisting pain, preoperative anxiety and depression, sexual satisfaction, dexmedetomidine administration, acute pain intensity at movement with 24 h.

**Table 2:** Univariate analysis of potential risk factors for CPSP at 3 months after hysterectomy

	No-CPSP 629	CPSP 241	χ^2^	P
Age				
< 45	91 (14.5%)	44 (18.3%)	1.924	0.382
45–54	344 (54.7%)	125 (51.9%)		
> 55	194 (30.8%)	72 (29.9%)		
ASA classification				
I-II	606 (92.3%)	225 (93.4%)	3.62	0.57
III-IV	23 (3.7%)	16 (6.6%)		
Education				
Illiteracy	63 (10.0%)	27 (11.2%)	0.593	0.743
Elementary education	424 (67.4%)	156 (64.7%)		
High school and above	142 (22.6%)	58 (24.1%)		
Employment				
Employed	272 (43.2%)	98 (40.7%)	2.306	0.316
Housewife	103 (16.4%)	50 (20.7%)		
Retired	254 (40.4%)	93 (38.6%)		
Preoperative pain				
Pelvic	46 (7.3%)	39 (16.2%)	74.432	< 0.001
Elsewhere	35 (5.65)	53 (22.0%)		
No	548 (87.1%)	149 (61.8%)		
Preoperative analgesic				
Paracetamol	27 (4.3%)	16 (6.6%)	6.39	0.09
NSAIDs	21 (3.3%)	14 (5.8%)		
Opioid	9 (1.4%)	6 (2.5%)		
None	572 (90.9%)	205 (85.1%)		
BMI				
<24.44	531 (84.45)	197 (81.7%)	1.052	0.591
24.44–28.08	87 (13.8%)	40 (16.6%)		
>28.08	11 (1.7%)	4 (1.7%)		
Smoking				
Yes	24 (3.8%)	11 (4.6%)	0.253	0.615
No	605 (96.2%)	230 (95.4%)		
Alcohol				
Yes	36 (5.7%)	9 (3.7%)	1.405	0.236
No	593 (94.3%)	232 (96.3%)		
Indicator for hysterectomy				
Myomas	433 (68.8%)	150 (62.2%)	6.073	0.415
Menorrhagia	23 (3.7%)	11 (4.6%)		
Dysmenorrhoea	11 (1.7%)	5 (2.1%)		
Cervical dysplasia	67 (10.7%)	39 (16.2%)		
Endometriosis	25 (4.0%)	9 (3.7%)		
Uterine prolapsed	42 (6.7%)	17 (7.1%)		
Adnexal mass	28 (4.5%)	10 (4.1%)		
Hypertension				
Yes	69 (11.0%)	33 (13.7%)	1.248	0.264
No	560 (89.0%)	208 (86.3%)		
Diabetes				
Oral medication or diet	38 (6.0%)	21 (8.7%)	2.005	0.367
On insulin	12 (2.0%)	4 (1.7%)		
No	579 (92.1%)	216 (89.6%)		
Coronary heart disease				
Yes	7 (1.1%)	2 (0.8%)	0.136	0.712
No	622 (98.9%)	239 (99.2%)		
Prior caesarean section				
Yes	33 (5.2%)	21 (8.7%)	3.598	0.058
No	596 (94.8%)	220 (91.3%)		
Prior laparotomy (Not CS)				
Yes	38 (6.0%)	23 (9.5%)	3.278	0.070
No	591 (94.0%)	218 (90.5%)		
Prior laparoscopy				
Yes	24 (3.8%)	14 (5.8%)	1.658	0.198
No	605 (96.2%)	227 (94.2%)		
Anxiety (HADS)				
Yes	88 (14.0%)	55 (22.8%)	9.893	0.002
No	541 (86.0%)	186 (77.2%)		
Depression(HADS)				
Yes	31 (4.9%)	22 (9.1%)	5.373	0.02
No	598 (95.1%)	219 (90.9%)		
Sexual satisfaction				
Very-moderately satisfied	337 (53.6%)	107 (44.4%)	16.166	< 0.001
Equally satisfied and dissatisfied	204 (32.4%)	73 (30.3%)		
Very-moderately dissatisfied	88 (14.0%)	61 (25.3%)		
Type of surgery				
lower abdominal	223 (35.5%)	86 (35.7%)	2.479	0.648
Pfannenstiel	158 (25.1%)	60 (24.9%)		
Vaginal	76 (12.1%)	37 (15.4%)		
LH	133 (21.1%)	47 (19.5%)		
LAVH	39 (6.2%)	11 (4.6%)		
Type of anesthesia				
TIVA	168 (26.7%)	74 (30.7%)	1.558	0.459
IA	95 (15.1%)	37 (15.4%)		
Epidural	366 (58.2%)	130 (53.9%)		
Postoperative analgesia				
PCEA	366 (58.2%)	130 (53.9%)	1.282	0.258
PCIA	263 (41.8%)	111 (46.1%)		
Dexmedetomidine				
Infusion(0.5 μg/kg/h)	109 (17.3%)	22 (9.1%)	10.16	0.006
Bolus(1 μg/kg)	104 (16.5%)	37 (15.4%)		
No	416 (66.1%)	182 (75.5%)		
Ketamine				
Bolus(1 mg/kg)	37 (5.9%)	11(4.6%)	0.581	0.446
No	592 (94.1%)	230 (95.4%)		
Acute pain intensity at rest with 24 h				
NRS < 3	511 (81.2%)	185 (76.8%)	2.182	0.14
NRS ≥ 3	118 (18.8%)	56 (23.2%)		
Acute pain intensity at movement with 24 h				
NRS < 3	464 (73.8%)	147 (61.0%)	13.595	< 0.001
NRS ≥ 3	165 (26.2%)	94 (39.0%)		
Blood loss				
< 400 ml	603 (95.8%)	228 (94.6%)	0.647	0.421
≥ 400 ml	26 (4.1%)	13 (5.4%)		
Duration of surgery				
< 2 h	582 (92.5%)	213 (88.4%)	3.802	0.051
≥ 2 h	47 (7.5%)	28 (11.6%)		
Blood infusion				
Yes	17 (2.7%)	9 (3.7%)	0.640	0.424
No	612 (95.7%)	232 (96.3%)		

As Table 3 shows, 12 variables were included in the subsequent multiple logistic regression models predicting CPSP at 3 months, because *P* < 0.25 for the comparisons between patients who did and did not develop chronic persistent pain. The independent predictors for CPSP following hysterectomy were indentified with preoperative pain, anxiety, depression, sexual dissatisfaction, postoperative acute pain intensity NRS ≥ 3 with 24 h at movement. Meanwhile, Intra-operative dexmedetomidine infusion with 0.5 μg/kg/h was associated with a decreased incidence rate of chronic post-hysterectomy pain.

**Table 3:** Logistic regression model for presence of CPSP at 3 months following hysterectomy

	Odds Ratio	95% Confidential Interval	*P* value
Preoperative pain			
Pelvic	3.531	2.163-5.763	< 0.001
Elsewhere	6.227	3.814-10.165	< 0.001
None	ref	ref	ref
Alcohol			
Yes	0.709	0.317-1.586	0.402
No	ref	ref	ref
Prior caesarean section			
Yes	1.634	0.863-3.093	0.132
No	ref	ref	ref
Prior laparotomy (Not CS)			
Yes	1.595	0.881-2.887	0.123
No	ref	ref	ref
Prior laparoscopy			
Yes	1.503	0.707-3.196	0.290
No	ref	ref	ref
Anxiety (HADS)			
Yes	2.071	1.363-3.147	0.001
No	ref	ref	ref
Depression(HADS)			
Yes	2.213	1.188-4.123	0.012
No	ref	ref	ref
Sexual satisfaction			
Very-moderate satisfied	0.742	0.497-1.107	0.144
Equally satisfied and dissatisfied	ref	ref	ref
Very-moderate dissatisfied	2.366	1.476-3.793	< 0.001
Dexmedetomidine			
Infusion(0.5 μg/kg/h)	0.461	0.283-0.753	0.002
Bolus(1 μg/kg)	0.813	0.538-1.23	0.327
No	ref	ref	ref
Acute pain intensity at rest with 24 h			
NRS < 3	ref	ref	ref
NRS ≥ 3	0.762	0.464-1.251	0.282
Acute pain intensity at movement with 24 h			
NRS < 3	ref	ref	ref
NRS ≥ 3	2.240	1.455-3.446	< 0.001
Duration of surgery			
< 2 h	ref	ref	ref
≥ 2 h	1.458	0.786-2.705	0.232

## Discussion

In our prospective cohort study conducted on 870 women who underwent hysterectomy, the incidence of CPSP was 27.7% at 3 months after surgery. Bransborg et al. [13] reported a pain prevalence of 31.9% 1 year after hysterectomy in Denmark in a nationwide questionnaire and database study, and then published a database survey on chronic pain after hysterectomy with a wide range incidence between 4.7 and 31.9% [1]. However, in another prospective multicenter cohort study in Netherlands, the prevalence of CPSP at 3 months after hysterectomy has been reported to be 10.2% [14]. The rationale of the lower incidence rate of CPSP was the diagnostic criteria used, and only those patients whose NRS ≥ 4 at follow-up after 3 months were identified to be experiencing CPSP. In our study, we diagnose CPSP in accordance with the IASP, and the incidence rate of CPSP is close to that in Bransborg report.

The incidence of neuropathic pain ranged widely after various types of surgery. The prevalence of probable or definite neuropathic pain among patients with chronic pain was 52-66% after thoracic surgery, 68-74% after breast surgery, 31-45% after hernia repair, and 6-9% after total hip and knee arthroplasty [15]. A multicenter cohort study including vaginal and abdominal hysterectomies found that 24 and 44% of patients with CPSP had neuropathic pain after vaginal and abdominal methods of hysterectomy, respectively [3]. In the current study, nearly half of the patients were assessed to be experiencing neuropathic pain. It has been proposed that different combinations of mechanisms involved in neuropathic and nociceptive pain might cause persistent pain after hysterectomy [16, 17], which may probably explain the high pavelance of neuropathic pain in our study.

We found most of patients with CPSP experienced mild pain with a low frequency. A few women reported moderate to severe pain. Pain is localized to the area of surgical incision and pelvis. Our study also found that CPSP had a slight negative impact on daily life of patients undergoing hysterectomy, particularly in mood and sleep.

Previous research has identified multiple risk factor for CPSP in different surgeries, including age, employment, BMI, education, smoking, preoperative co-morbidities [2, 18–20]. No significant differences in above mentioned variables were observed between treatments in our study.

Previous surgery has been recognized as a risk factor for development of chronic pain after hysterectomy. In present study, prior surgery including cesarean section, laparoscopy, and laparotomy were associated with chronic pain in univariate but not in multiple logistic regression analyses.

A large body of evidence suggests that the presence of preoperative pain in patients may be related to CPSP [21–24]. Pre-surgical pain predisposes to central sensitization, which has been considered as the underlying mechanism of CPSP. In the current study, we found that the presence of preoperative pain was a risk factor for CPSP after hysterectomy, which supported the findings of Pinto et al. [2].

Psychological factors including anxiety and depression, play a critical role in the development of CPSP [25]. A series of studies published by Brondsborg et al. [13] showed that preoperative psychological distress was associated with CPSP following hysterectomy [1, 13, 26]. One of the most recent systematic reviews targeting the role of psychosocial predictors of CPSP demonstrates a significant positive association between preoperative anxiety as well as pain catastrophizing and CPSP after hysterectomy [27]. Likewise, pre-surgical depression was considered to relate to an elevated incidence of chronic pain in a prospective cohort study concerning hysterectomy [28]. In the present study, we also found that preoperative anxiety and depression as predisposing factors for the development of chronic pain after hysterectomy.

The effect of hysterectomy on female sexual function has been well studied [29, 30]. On the contrary, the influence of pre-surgical sexual satisfaction on hysterectomy remains unclear. To our knowledge, we firstly investigated sexual satisfactions of the participants, and found that a very-moderate sexual satisfaction before surgery was an independent predictive factor for CPSP after hysterectomy. Moreover, a preoperative very-moderate sexual dissatisfaction was always associated with pre-surgical pelvic pain, which could explain our findings.

Surgical approach associated with nerve damage has been considered as risk factor for CPSP in many operations. Laparoscopy may minimize the trauma and reduce the risk of nerve injury, leading to a decreased incidence of CPSP, which had been verified in laparoscopic hernia repair [31]. Pinto et al. [2] also found vaginal route or laparoscopic approaches of hysterectomy was associated with a lower incidence of CPSP as compared to abdominal hysterectomy, and proposed of the type of hysterectomy as predictive factor of persistent postsurgical pain 4 months following hysterectomy. On the contrary, we did not find contribution of surgical approach to the development of CPSP in our study, which was consistent with the findings of Brandsborg [13] and Theunissen [14]. A cross-sectional study to compare CPSP after robot-assisted laparoscopic hysterectomy and abdominal hysterectomy showed that the type of surgical approach did not influence the development of CPSP following hysterectomy [32]. The result supported our opinion from another point of view.

Postoperative epidural analgesia was showed to be related to a reduced incidence of CPSP after abdominal surgery [33]. In our study, we used both epidural analgesia with morphine and intravenous analgesia with sufentanil for treatment of postoperative pain. However, we did not find significant difference in post-hysterectomy analgesia approach between CPSP and No-CPSP, suggesting that the type of postoperative analgesia would not determine the development of CPSP after hysterectomy.

Substantial evidence indicates that intensity of acute postoperative pain was associated with the development of CPSP following hysterectomy [2, 14, 19, 26]. As stated previously, acute postoperative pain NRS ≥ 4 both at rest and at movement were associated with CPSP in univariate analysis. However, only pain NRS ≥ 4 at movement was related to CPSP in multiple logistic regression analyses. This result was in agreement with Brandsborg and Theunissen, indicating that postoperative pain management, but not postoperative analgesia approach, plays a critical role in prevention of the development of CPSP.

The effect of anesthetics on development of CPSP is still debatable. Ketamine, an NMDA receptor antagonist, usually used as an adjunct peri-operative analgesic, could reduce the rate of CPSP via a mechanism of preventing peripheral and central sensitizations [34]. Administration ketamine in multi-modal analgesic model did decrease the prevalence of CPSP [35, 36]. However, in a systematic review and meta-analysis of ketamine for the prevention of persistent post-surgical pain, ketamine did not provide a significant reduction of CPSP at 3 and 6 months [5]. In our study, we used ketamine with 1 mg/kg bolus as an analgesic adjuvant during hysterectomy and did not find the effectiveness of ketamine use on the prevention of CPSP following hysterectomy. Dexmedetomidine, an alpha-2 agonist with analgesic, sedative-hypnotic, and sympatholytic properties, has been investigated to find its effect on acute post hysterectomy pain [37–39]. In a recent randomized clinical trial, pre-emptive dexmedetomidine reduced the incidence of chronic post-thoracotomy pain after coronary artery bypass grafting [40]. In the present study, there were two ways of intra-operative dexmedetomidine administration, infusion with 0.5 μg/kg/h from anesthesia induction to extubation at the end of surgery and bolus with 1 μg/kg before incision. We also found that intra-operative use of dexmedetomidine infusion with 0.5 μg/kg/h could decrease the rate of development of CPSP after hysterectomy. It has been reported that dexmedetomidine decrease sympathetic tone and cytokines release to surgical stress [41], which could attenuate peripheral sensitization and central sensitization associated with long-term potentiation and CPSP. Furthermore, in an experimental study, dexmedetomidine modified descending control of nociception by decreasing the threshold for descending inhibition and/or increasing the threshold for descending facilitation [42]. These results may explain that our clinic practice with dexmedetomidine infusion with 0.5 μg/kg/h decrease the rate of development of CPSP after hysterectomy.

Our study has some limitations that need to be addressed. First of all, the study relied on the subjective self-report, which might be influenced by the psychological and mental status of patients. Second limitation was the data excluded, which could impact the accuracy of the results. Another defect was that most of patients were local residents, therefore, the results of the current study cannot be generalized to women population in China. Lastly, in some cases, economic factors may influence the choice of surgical approach.

Despite these limitations, one advantage of this study was the prospective design. Patients in our study were asked to report pain at interviews, rather than recalling previous pain experiences in retrospective studies. Another merit of this study was the diversity of the treatments, which allows investigating multiple variables.

## Conclusion

In summary, 28 % of patients after hysterectomy in southern Jiangsu china had CPSP with 92% of those women describing it as mild. Preoperative anxiety and depression, poor sexual satisfaction, presence of pre-surgical pain, and acute postoperative pain on movement are risk factors for development of CPSP in women in China. Intra-operative dexmedetomidine infusion with 0.5 μg/kg/h is associated with a decreased incidence of chronic post-hysterectomy pain. These results suggest that preventive strategies should be targeted at preoperative psychosocial care and postoperative pain management. A further controlled prospective randomized trial on dexmedetomidine is needed.

## Abbreviations

BMI: Body Mass Index
BPI-SF: Brief Pain Inventory-Short Form
CPSP: Chronic Post-Surgical Pain
DN-4: Douleur Neuropathic 4-Questionnaire
FSFI: Female Sexual Function Index
HAD-A: Hospital Anxiety and Depression Scale- Anxiety subscale
HAD-D: Hospital Anxiety and Depression Scale- Depression subscale
HADS: Hospital Anxiety and Depression Scale
IA: Inhalation Anesthesia
IASP: International Association for the Study of Pain
ICD-9: International Classification Diseases-9
LAVH: Laparoscopic Assisted Vaginal Hysterectomy
LH: Laparoscopic Hysterectomy
MMPI: Minnesota Multiphasic Personality Inventory
MSPQ: Modified Somatic Perception Questionnaire
NMDA: N-methyl-D-aspartate
NP: Neuropathic Pain
NRS: Numerical Rating Scale
SPSS: Statistical Package for Social Science
STAI: State-Trait Anxiety Inventory
TIVA: Total IntraVenous Anesthesia
